# Supplementary figures and images for: Seasonal patterns of ecological uniqueness of anuran metacommunities along different ecoregions in Western Brazil
Source: PLoS One. 2020 Sep 24;15(9):e0239874. doi: 10.1371/journal.pone.0239874 (PMC7514074; doi:10.1371/journal.pone.0239874)

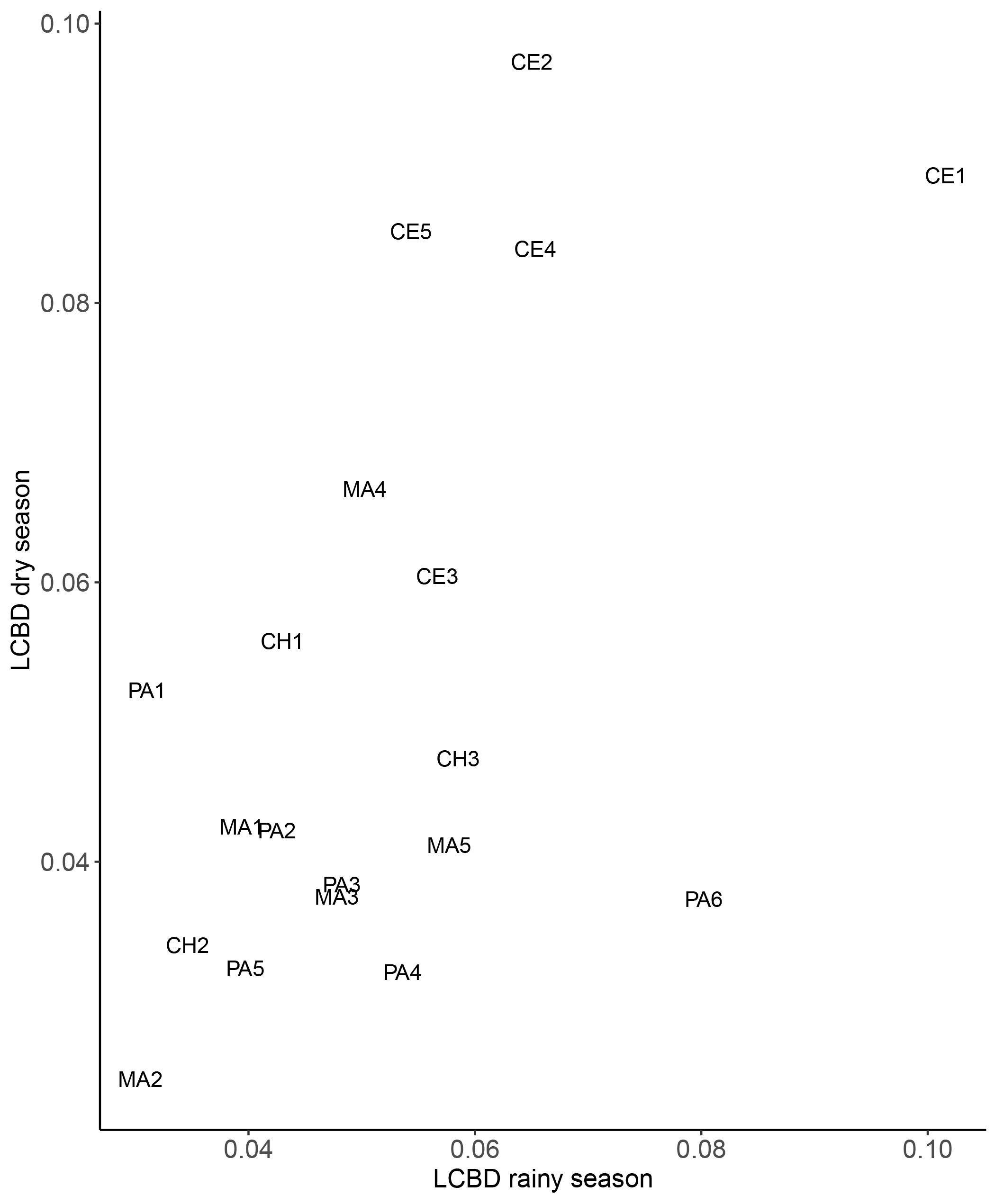

Supplement: S1 Fig — Sites abbreviation can be seen in the S1 Table. (JPG) [file pone.0239874.s001.jpg]

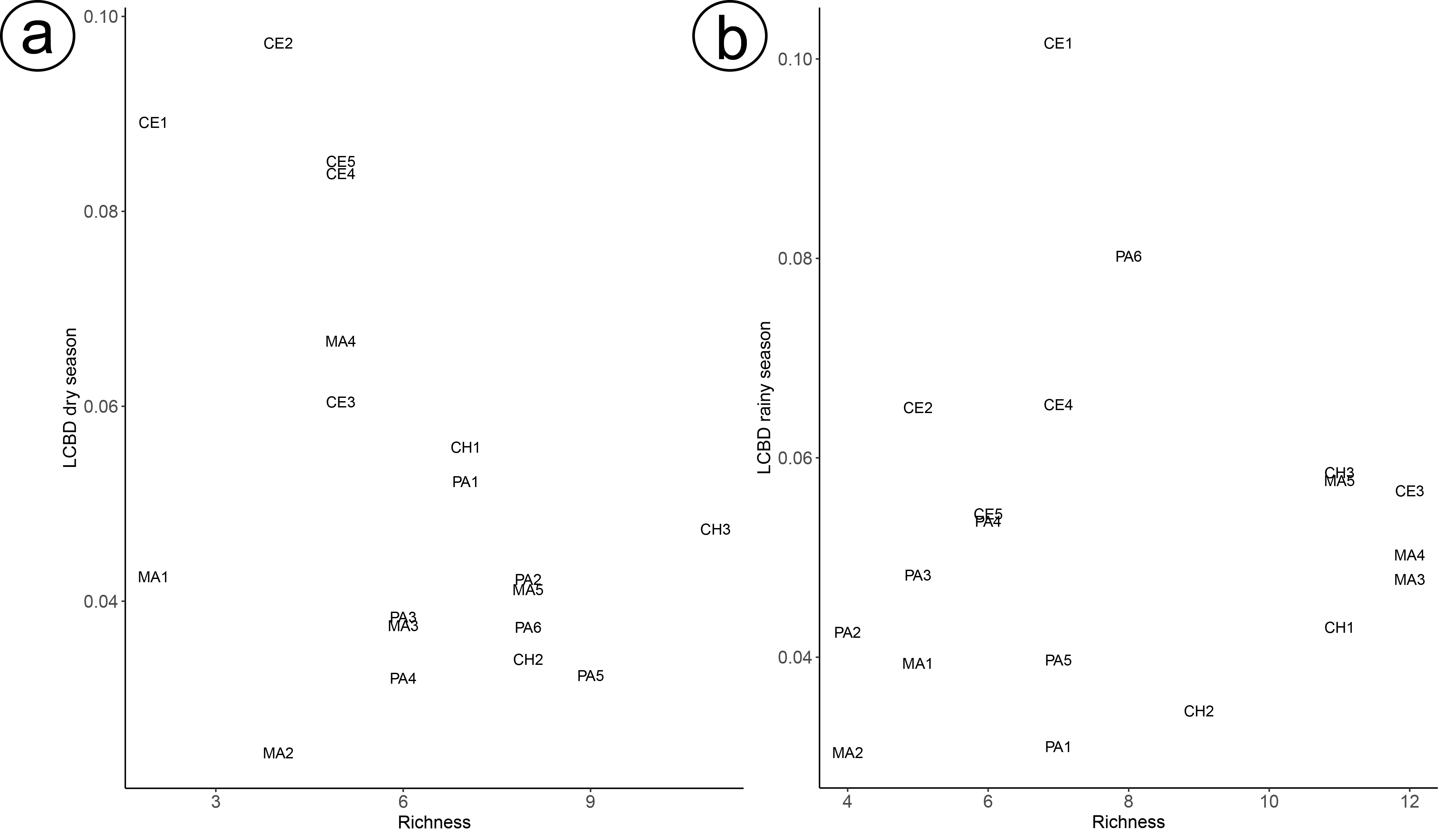

Supplement: S2 Fig — Pearson correlation between richness and LCBD values during dry (a) and rainy seasons (b). To studied sites abbreviation see S1 Table. (JPG) [file pone.0239874.s002.jpg]
