# Supplementary material for: Seasonal patterns of ecological uniqueness of anuran metacommunities along different ecoregions in Western Brazil
Source: PLoS One. 2020 Sep 24;15(9):e0239874. doi: 10.1371/journal.pone.0239874 (PMC7514074; doi:10.1371/journal.pone.0239874)
Supplement: S1 Table — (DOCX) [file pone.0239874.s003.docx]

**S1 Table.** Ponds sampled during the years of 2017 and 2018 in West Brazil.

| **Name site** | **Site** | **Formation** | **Lat** | **Long** | **Pond área (m²)** | **Wet richness** | **Dry richness** | **Sample coverage** |
| --- | --- | --- | --- | --- | --- | --- | --- | --- |
| Brejo Bonito | CE1 | Cerrado | -20.5377 | -54.7548 | 6617 | 7 | 2 | 61.15 |
| Camapuã | CE2 | Cerrado | -19.0142 | -53.8591 | 866 | 5 | 4 | 78.41 |
| Mimosa 01 | CE3 | Cerrado | -20.9659 | -56.524 | 1355 | 12 | 5 | 100.00 |
| Mimosa 02 | CE4 | Cerrado | -20.9685 | -56.5211 | 1018 | 7 | 5 | 73.09 |
| Taquari | CE5 | Cerrado | -18.1571 | -53.413 | 5770 | 6 | 5 | 89.21 |
| Chaco 01 | CH1 | Chaco | -21.6929 | -57.7169 | 1736 | 11 | 7 | 53.09 |
| Chaco 02 | CH2 | Chaco | -21.6065 | -57.8163 | 802 | 9 | 8 | 94.17 |
| Chaco 03 | CH3 | Chaco | -21.71 | -57.7209 | 1146 | 11 | 11 | 92.64 |
| Três Lagoas 01 | AF1 | Atlantic Forest | -20.7513 | -51.6544 | 839 | 5 | 2 | 73.17 |
| Três Lagoas 02 | AF2 | Atlantic Forest | -20.7727 | -51.7158 | 2888 | 4 | 4 | 83.57 |
| Ivinhema 01 | AF3 | Atlantic Forest | -22.9218 | -53.6571 | 1809 | 12 | 6 | 60.48 |
| Ivinhema 02 | AF4 | Atlantic Forest | -22.9008 | -53.7471 | 652 | 12 | 5 | 60.30 |
| Ivinhema 03 | AF5 | Atlantic Forest | -22.889 | -53.6439 | 797 | 11 | 8 | 65.10 |
| Barranco Alto 01 | PA1 | Pantanal | -19.5724 | -56.1548 | 2061 | 7 | 7 | 100.00 |
| Barranco Alto 02 | PA2 | Pantanal | -19.5719 | -56.144 | 4250 | 4 | 8 | 100.00 |
| BEP 01 | PA3 | Pantanal | -19.5752 | -57.0217 | 1116 | 6 | 5 | 53.25 |
| BEP 02 | PA4 | Pantanal | -19.5765 | -57.0187 | 434 | 6 | 6 | 97.33 |
| Baía Negra 01 | PA5 | Pantanal | -19.0222 | -57.5106 | 6670 | 7 | 9 | 74.47 |
| Baía Negra 02 | PA6 | Pantanal | -19.0184 | -57.5564 | 4052 | 8 | 8 | 75.30 |
