# Supplementary material for: Seasonal patterns of ecological uniqueness of anuran metacommunities along different ecoregions in Western Brazil
Source: PLoS One. 2020 Sep 24;15(9):e0239874. doi: 10.1371/journal.pone.0239874 (PMC7514074; doi:10.1371/journal.pone.0239874)
Supplement: S2 Table — All temperature and precipitation values were extracted from BioClim (http://worldclim.org/current) for each studied community. All values were averaged over the surrounding 2km to help buffer uncertainty in the reported locations. Variables indicates the name of the climatic variable in the respective date source. (DOCX) [file pone.0239874.s004.docx]

**S2 Table.** Raw climatic variables. All temperature and precipitation values were extracted from BioClim (http://worldclim.org/current) for each studied community. All values were averaged over the surrounding 2km to help buffer uncertainty in the reported locations.

| **Bioclimatic variables** | **Units** | **Description** |
| --- | --- | --- |
| BIO1 | C^0^× 10 | Annual Mean Temperature |
| BIO2 | C^0^× 10 | Mean Diurnal Range |
| BIO3 | ratio | Isothermality (BIO2/BIO7) (* 100) |
| BIO4 | stdev ×100 | temperature seasonality |
| BIO5 | C^0^× 10 | Max Temperature of Warmest Month |
| BIO6 | C^0^× 10 | Min temperature of coldest month |
| BIO7 | C^0^× 10 | Temperature Annual Range (BIO5-BIO6) |
| BIO8 | C^0^× 10 | Mean Temperature of Wettest Quarter |
| BIO9 | C^0^× 10 | Mean Temperature of Driest Quarter |
| BIO10 | C^0^× 10 | Mean Temperature of Warmest Quarter |
| BIO11 | C^0^× 10 | Mean Temperature of Coldest Quarter |
| BIO12 | mm | Annual Precipitation |
| BIO13 | mm | Precipitation of Wettest Month |
| BIO14 | mm | Precipitation of Driest Month |
| BIO15 | coef var | Precipitation Seasonality |
| BIO16 | mm | Precipitation of Wettest Quarter |
| BIO17 | mm | Precipitation of Driest Quarter |
| BIO18 | mm | Precipitation of Warmest Quarter |
| BIO19 | mm | Precipitation of Coldest Quarter |
