# Supplementary material for: Seasonal patterns of ecological uniqueness of anuran metacommunities along different ecoregions in Western Brazil
Source: PLoS One. 2020 Sep 24;15(9):e0239874. doi: 10.1371/journal.pone.0239874 (PMC7514074; doi:10.1371/journal.pone.0239874)
Supplement: Attachment — Submitted filename: Answers to reviewers comments.pdf [file pone.0239874.s006.pdf]

Rebuttal letters referring to ms PONE-D-20-20490R1

Dear Dr. Ricardo Bomfim Machado

Academic Editor of PLoS ONE Journal

Thank you for the invitation to submit a new revised version of our manuscript. We appreciate the constructive criticisms and suggestions by the editor and referee. We considered all points raised by the editor and reviewer, and carefully addressed them as described below.

In summary, we modified our title based on the reviewer suggestion and we improved our hypothesis about the restriction of water availability involving Cerrado and Chaco ecoregions.

We believe that this new version of the manuscript incorporates all main aspects pointed out by the reviewers, and hope that it meets the high standards for publication in PLoS ONE.

Reviewer #2

1. Title. The title is not accurate enough because it leads the reader to a broader scope of ecoregion of Brazil as it is: “Seasonal patterns of ecological uniqueness of anuran metacommunities along different Brazilian ecoregions”, but the sampling of these in parts of these ecoregions was limited to Mato Grosso do Sul state. Brazil has more than 20 ecoregions distributed across 6 main vegetation domains. In this paper, only 4 were sampled and again I am not totally secure that those ponds on ecotones between two different ecoregions belong to one or another. The number of ecoregions sampled may be due to some disparities in map projections and sampling coordinates. Even if such problems are overcome, I would still suggest that the title be adjusted to: “Seasonal patterns of ecological uniqueness of anuran metacommunities along different ecoregions in Western Brazil” in order to describe the paper content more accurately.

R: We are grateful for this suggestion. We modified the title as recommended. Now it reads: “Seasonal patterns of ecological uniqueness of anuran metacommunities along different ecoregions in Western Brazil”.

2. Hypothesis/Prediction. The authors state that “LCBD patterns would differ between seasons due to the restriction of water availability in dry compared to rainy seasons. This would filter species in naturally dry ecoregions, such as Cerrado, where water availability is a constraint for many species [37], leading to more unique communities.” Later, the authors state that “The Chaco ecoregion receive around >700 mm per year of rainfall”, which is the lowest total amount of rain among ecoregions

(Atlantic Forest: 1000 mm/y; Cerrado: 800-2000 mm/y; Pantanal: 1089 mm/y). Besides, Chaco comprises xerophytic vegetation. Therefore, this hypothesis/prediction is not supported by the description of ecoregions and I had a hard time understanding this hypothesis/prediction. I think the authors need to be clearer in what sense they assess/characterize dry conditions among ecoregions to support the hypothesis/prediction.

R: We agree with your comment. First, these values of rainfall are the range for each ecoregion. For example, Cerrado is the second larger ecoregion in Brazil (and in South America) and is bordered by many ecoregions, including Atlantic Forest, Pantanal, Amazônia, and Caatinga, justifying this high range in rainfall. Also, both Cerrado and Chaco are considered seasonally dry tropical forest (Pennington et al. 2009), meaning that rainfall is less than c. 1800mm per year, with a period of at least 5-6 months receiving less than 100mm (Pennington et al. 2009). This seasonality of Cerrado and Chaco justify our hypothesis of higher LCBD values for Cerrado and Chaco (see the small change we made below). We updated the mean annual precipitation in the methods including values for the studied region (Fick & Hijmans 2017) and we also included the mean precipitation seasonality for each ecoregion (lines 154, 162, 166-169, 174). Despite Cerrado showed the highest and Chaco the least mean annual precipitation, the values between ecoregions are not so different. On the other hand, Cerrado is the second ecoregion in terms of seasonality and Chaco is the third. Although Pantanal had the highest seasonality, water is more available in this ecoregion due to large rivers (e.g. Rio Paraguai), and many natural lakes, known locally as baías. This would provide refuges for anuran species in the dry season, different from the Cerrado ecoregion. Our point here is that the ecoregions did not greatly vary in terms of rainfall, but they did in terms of seasonality, which may influence water availability for anurans.

We modified our hypothesis to (lines 127- 141): "We expected that LCBD would differ among ecoregions for the dry season, and no difference would be found in LCBD for the rainy season. This expectation is based on the low water availability in dry season compared to the rainy season, when all ecoregions tended to be equal in terms of water availability. This water restriction in the dry season would filter species in naturally seasonally dry ecoregions, such as the Cerrado and Chaco [37], where water availability is a constraint for many species in the dry season [37], leading to more unique communities. We also expected that this filter would be more intensive in the Cerrado because this ecoregion is not close to floodplains that may maintain water availability during the dry season. The Chaco region is close to the Pantanal and both occupy the area under influence of Paraguay Basin flood pulses, which would provide water to anuran reproduction throughout the year. In this way, we expected that the Cerrado ecoregion would have higher values of LCBD compared to other ecoregions in the dry season. We also hypothesized that LCBD variation would be driven by environmental variables in the dry and rainy seasons, but the total amount of variation would be higher in the dry season".

We also added in methodology the following information (lines 167-169): "The Cerrado and Chaco ecoregions are considered seasonally dry tropical forest, meaning that rainfall is less than c. 1800mm per year, with a period of at least 5-6 months receiving less than 100mm [37]."

Lines 187-190: “Cerrado and Pantanal ecoregion show the higher values of precipitation seasonality (55.54 and 59.01 coefficient of variation, respectively) in relation to Atlantic Forest (46.64 coefficient of variation) and Chaco (45.45 coefficient of variation) [42].”

References

Fick SE, Hijmans RJ. WorldClim 2: new 1-km spatial resolution climate surfaces for global land areas. Int J Climatol. 2017;37: 4302–4315.

Pennington RT, Lavin M, Oliveira-Filho A. Woody Plant Diversity, Evolution, and Ecology in the Tropics: Perspectives from Seasonally Dry Tropical Forests. Annu Rev Ecol Evol Syst. 2009;40: 437–457. doi:10.1146/annurev.ecolsys.110308.120327
